# Aquatic macroinvertebrate assemblages in rivers influenced by mining activities

**DOI:** 10.1038/s41598-022-06869-2

**Published:** 2022-02-25

**Authors:** Axel Eduardo Rico-Sánchez, Alexis Joseph Rodríguez-Romero, Jacinto Elías Sedeño-Díaz, Eugenia López-López, Andrea Sundermann

**Affiliations:** 1 Programa de Hidrociencias, Colegio de Postgraduados, Carr. México-Texcoco 36.5 km, Montecillo, Texcoco, Estado de México C.P. 56230 Mexico; 2 Laboratorio de Evaluación de la Salud de los Ecosistemas Acuáticos, Escuela Nacional de Ciencias Biológicas, Instituto Politécnico Nacional, Prolongación de Carpio y Plan de Ayala S/N, Col. Santo Tomás, Miguel Hidalgo, Mexico City, C.P. 11340 Mexico; 3 Coordinación Politécnica Para la Sustentabilidad, Instituto Politécnico Nacional, Av. Instituto Politécnico Nacional s/n, Esq. Wilfrido Massieu, Col. San Pedro Zacatenco, Gustavo A. Madero, Mexico City, C.P. 07738 Mexico; 4 Department of River Ecology and Conservation, Senckenberg Research Institute and Natural History Museum Frankfurt, Gelnhausen, Germany; 5 Department Aquatic Ecotoxicology, Goethe University Frankfurt am Main, Faculty of Biological Sciences, Frankfurt am Main, Germany

**Keywords:** Biodiversity, Community ecology, Conservation biology, Ecology, Ecology, Environmental sciences, Limnology

## Abstract

Mining is one of the major pollution sources worldwide, causing huge disturbances to the environment. Industrial and artisanal mining activities are widespread in Mexico, a major global producer of various metals. This study aimed to assess the ecological impairments resulting from mining activities using aquatic macroinvertebrates assemblages (MA). A multiple co-inertia analysis was applied to determine the relationships between environmental factors, habitat quality, heavy metals, and aquatic macroinvertebrates in 15 study sites in two different seasons (dry and wet) along two rivers running across the Central Plateau of Mexico. The results revealed three contrasting environmental conditions associated with different MAs. High concentrations of heavy metals, nutrients, and salinity limit the presence of several families of seemingly sensitive macroinvertebrates. These factors were found to influence structural changes in MAs, showing that not only mining activities, but also agriculture and presence of villages in the basin, exert adverse effects on macroinvertebrate assemblages. Diversity indices showed that the lowest diversity matched both the most polluted and the most saline rivers. The rivers studied displayed high alkalinity and hardness levels, which can reduce the availability of metals and cause adverse effects on periphyton by inhibiting photosynthesis and damaging MAs. Aquatic biomonitoring in rivers, impacted by mining and other human activities, is critical for detecting the effect of metals and other pollutants to improve management and conservation strategies. This study supports the design of cost-effective and accurate water quality biomonitoring protocols in developing countries.

## Introduction

Mining is an important pollution source worldwide, causing huge disturbances to aerial, terrestrial, and aquatic ecosystems^1–3^. Particularly in Latin America, mining activities produce metal pollution that is among the main stressors of aquatic ecosystems^4,5^. Additional significant sources of pollution in natural water bodies include agriculture (a key driver of deforestation, causing changes in land cover and use) and the massive use of agrochemicals^6^. Furthermore, industry and human settlements discharge wastewater containing complex mixtures of pollutants^7^. All these stressors cause the degradation of water quality in rivers, with adverse effects on aquatic life—particularly in tropical regions where human populations are experiencing an accelerated growth^8^. Mexico is the second most populated country in Latin America^9^ and one of the most biodiverse countries worldwide^10^. However, Mexico faces several serious pollution challenges from anthropogenic activities such as mining and agriculture, which affect biodiversity and ecosystems^10,11^. The country is the largest producer of silver in the world and a major global producer of gold, copper, and zinc, among other minerals^12^. Industrial and artisanal mining are widespread in the country, including opencast mining and mercury extraction, the latter associated with the use of mercury for gold amalgamation^13^. Artisanal mercury extraction is a prevalent activity in developing countries^14,15^.

Efforts to protect and preserve biological diversity are usually focused on protected natural areas (PNAs), which are the cornerstone of conservation strategies worldwide^16^. PNAs are especially important for being home to a high number of endemic species and a high biodiversity. However, legal mining operations are allowed in some of them^11^, in addition to mines that are either unlicensed or that were established before the government declared these areas as PNAs. To date, the federal government has decreed 182 PNAs to preserve the biological diversity of Mexico. PNAs in México harbor a high diversity of various taxonomic groups^17^, most of which are not systematically monitored today; this is especially true for freshwater species^18^. Nowadays, although PNAs are subject to a conservation protocol, these areas face several challenges in Mexico, as activities such as mining, deforestation, and agriculture are currently in place and cause adverse effects^19,20^. Recent studies by Armendáriz-Villegas et al.^11^ in Mexican PNAs, including the Sierra Gorda Biosphere Reserve (SGBR), have identified industrial and artisanal mining activities (exploration and exploitation) as important disturbance factors. There are more than 140 active mines in the Sierra Gorda region (Central México), 60% of which are located inside the SGBR polygon (mostly artisanal mining)^21^; however, the impacts of mining on aquatic life in PNAs have not been documented until now. Neither the detection of metals in water nor the impact of metals on aquatic ecosystems are systematically monitored in Mexico. Furthermore, biomonitoring is not part of government programs for environmental protection in Mexico to date.

Macroinvertebrates are recognized as the most suitable organisms for biomonitoring as they have low mobility, are in contact with both sediments and the water column, thus being exposed to pollutants in both compartments, and display a wide range of tolerance to contaminants (including from highly sensitive species to species that are very tolerant to polluted conditions)^22,23^. Thus, environmental disturbances can modify the structure of macroinvertebrate assemblages in response to several stressors. These modifications include changes in species composition and abundance in impacted areas with the predominance of tolerant species, while sensitive species occur only in areas with minimally disturbed conditions or negligible impacts^24^. Nevertheless, the composition of aquatic macroinvertebrate assemblages in Mexican PNAs has been little studied and scarce information is available on the habitat requirements of the aquatic macroinvertebrates or their particular responses to different stressors. Some experimental studies about the impact of heavy metals on the distribution and diversity of macroinvertebrates have been carried out^25–27^ and, in some cases, assemblage structure was included^26,28,29^. However, additional information is needed to fully understand the response of macroinvertebrates to heavy metals and other minerals from geological strata to assess the disturbances caused by human impacts on aquatic wildlife, especially in a PNA affected by mining activities^30^.

Therefore, this study aimed to identify how anthropogenic pollutants, particularly those associated with mining (metals), agriculture (nutrients), and villages (wastewater), affect aquatic macroinvertebrate assemblages in rivers with a high mineral content and different metal composition running across a PNA in Central Mexico. We first assessed the biological diversity of macroinvertebrates inhabiting the rivers in the study area and then contrasted the aquatic macroinvertebrate assemblages living in areas with high heavy metal levels and human impact versus assemblages in areas with low concentrations of heavy metals and minor human activities, identifying the patterns of aquatic macroinvertebrates associated with the variation in metal composition in the sites studied. As the SGBR is affected by mining activities, we emphasized the evaluation of heavy metals and taxa identification that might indicate inorganic pollution. In addition, we examined the impact of organic pollution on aquatic macroinvertebrate assemblages.

## Results

### Aquatic macroinvertebrate assemblages

**Figure 1:**
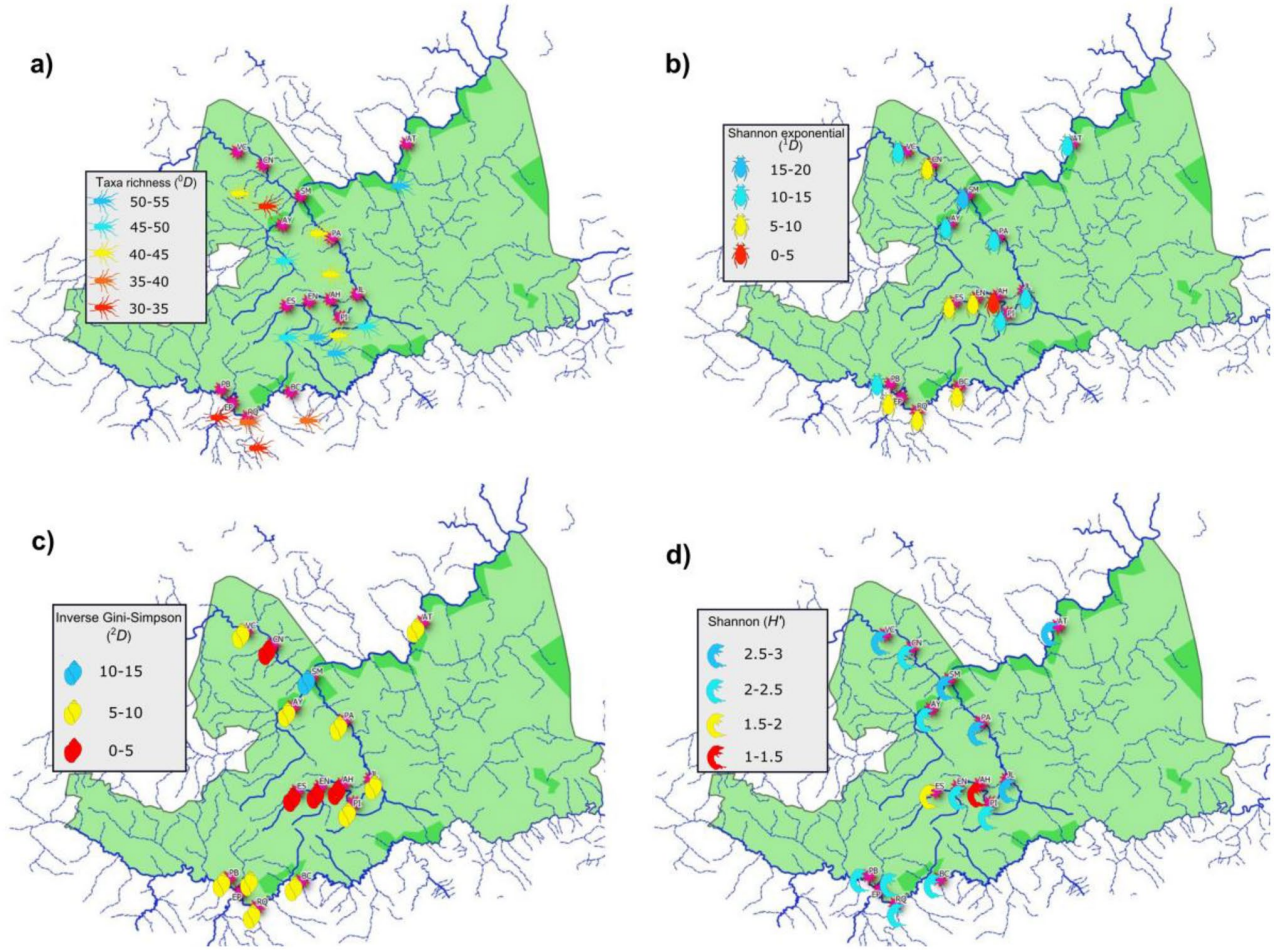
Heatmap of diversity indices for the study sites in the SGBR: **(a)** taxa richness, **(b)** Shannon exponential index, **(c)** Inverse Gini-Simpson index, **(d)** Shannon index. The regional map was generated using the vectorial layers freely available from National Institute of Statistics, Geography and Informatics (https://www.inegi.org.mx/temas/mapadigital) and National Commission of Protected Natural Areas (http://sig.conanp.gob.mx/website/pagsig/info_shape.htm. All layers were processed with the open source software geographic information system QGIS 3.18 (QGIS is open source software available under the terms of the General Public License (GNU) meaning that source codes can be downloaded through tarballs or the git repository). Study site points were downloaded from a hand-held GPS (Monterra^®^|Garmin) before being digitized and uploaded as shapefiles. Sampling points and legend layouts were edited using open source software available at: https://inkscape.org. The sampling points show diversity calculated from iNEXT package (; Hsieh, T.C., Ma, K. H. & Chao, A. (2016) iNEXT: An R package for interpolation and extrapolation of species diversity) and processed using R Core Team version 3.1.0. (A language and environment for statistical computing. R Foundation for Statistical Computing, Vienna, Austria; https://www.R-project.org/).

A total of 77,000 specimens in 93 families, 23 orders, and six phyla were identified in 15 study sites and two seasons. Taxonomic richness (^0^*D*) was lowest (37 ± 4 families) in the Extoraz River (sites PB, EP RQ, BC) and highest (50 ± 1.7 taxa) in the Escanela-Jalpan and Santa María Rivers (Fig. 1 and Supplementary Table S1). There were statistically significant differences between the Extoraz and Escanela-Jalpan Rivers (Tukey HSD; *p* ≤ 0.05). The Shannon diversity index (*H’*) showed minor differences between sites (2.25 ± 0.42). The lowest diversity values (1.63 ± 0.5) were recorded in the Escanela River (sites ES, EN, and, particularly, AH) (Fig. 1 and Supplementary Table S1) and the highest (2.67 ± 0.1) in the Santa María River (sites SM, AT). The exponential of Shannon’s diversity index (^1^*D*) showed the lowest values in the Extoraz (except for site PB, where diversity was high) and Escanela Rivers (sites EN, ES, and particularly site AH, where the lowest value was recorded). In contrast, the highest values were recorded in the Jalpan and Santa María Rivers (sites PI, JL, PA, AY, and SM) (Fig. 1 and Supplementary Table S1). The dominance index (the inverse of the Gini-Simpson’s ^2^*D*) was lowest in the Escanela and Concá Rivers, where dominance was high. The Extoraz River showed intermediate ^2^*D* values (in contrast with low ^0^*D* values), indicating moderate dominance of a few taxa (Diptera). High ^2^*D* values were observed at site SM (Santa María River), where the abundance of the different taxa showed high evenness (see Fig. 1 and Supplementary Table S1).

### Environmental factors

A few environmental factors showed a high degree of collinearity (Spearman’s *rho* ≥ 0.80, Supplementary Table S2). Total coliforms, color, electrical conductivity, sulfates, total suspended solids, turbidity, and iron were excluded from further analyses because they were correlated with fecal coliforms, ammonium, salinity, and aluminum, which were kept for subsequent analysis. As shown by their hardness, alkalinity, chloride, and salinity values (Table 1), some rivers (Extoraz and Concá) are highly mineralized. A high concentration of fecal coliforms and high oxygen demand (BOD5) revealed domestic wastewater pollution in the Escanela-Jalpan River. Other sites (e.g., sites on the Extoraz and Concá Rivers) are degraded by nutrients from various sources. Overall, all study sites were well-oxygenated (Table 1). High heavy metal concentrations were found in all SGBR rivers. The rank of predominance of heavy metals in the rivers was as follows: Extoraz basin: Al > Fe > Zn > Cd > As > Hg > Mn > Sb > Co > Cr > Cu; Escanela-Jalpan: Zn > Fe > Al > As > Cd > Hg > Sb > Cu > Cr > Co > Mn; Ayutla: As > Fe > Al > Zn > Sb > Hg > Cd > Co > Cr > Cu > Mn; Concá, Al > Fe > Hg > Zn > As > Cd > Sb > Co > Cr > Cu > Mn; and Santa María: Al > Fe > Zn > Cd > As > Sb > Hg > Co > Cr > Cu > Mn (See Supplementary Table S3).

**Table 1:** Selected environmental factors for further analysis at 15 study sites.

Factor	Min	Max	Mean	SE
Alkalinity	290	381	335	8.07
Cloride	10.43	37.86	16.13	1.83
Fecal coliforms	25	780	338	60.92
BOD5	1.19	3.61	1.92	0.17
Hardness	93.75	264.13	129.97	11.60
NH3	0.09	0.66	0.35	0.06
NO2	0.001	0.03	0.01	0.00
NO3	0.8	1.51	1.31	0.07
TN	2.73	6.49	1.35	0.32
DO	7.41	10.13	8.74	0.21
O-PO4	0.13	0.61	0.33	0.04
pH	7.9	8.5	8.1	0.04
TP	0.25	1.68	0.69	0.11
Salinity	0.15	0.51	0.23	0.02
Water temperature	15.9	23.3	20.5	0.63
Air temperature	19.9	28.8	23.5	0.69
Discharge	0.31	18.43	4.5	1.30
Habitat assessment	7.8	16.9	13.1	0.66
**Epifaunal avaible substrate cover**	5	20	14	0.95
**Substrate heterogeneity**	5	20	14	0.87
**Velocity**	3	20	12	1.04
**Sediment deposition**	8	20	14	0.72
**Channel flow status**	3	20	14	0.91
**Chann. alter**	5	20	16	0.88
**Channel sinuosity**	5	20	12	0.76
**Bank stability**	4	20	11	0.96
**Bank vegetative protection**	2	20	11	0.95
**Riparian vegetation width**	1	20	11	1.10
Al	0.001	2.179	0.292	0.141
As	0.002	0.085	0.026	0.006
Cd	0.001	0.277	0.028	0.018
Hg	0.003	0.058	0.013	0.005
Sb	0.001	0.031	0.007	0.002
Zn	0.005	0.538	0.082	0.036
Cr	0.001	0.004	0.001	0.000
Cu	0.001	0.012	0.002	0.001

Bold factors are those included in the visual-based habitat assessment.

*BOD*_5_ biochemical oxygen demand of 5 days, *TN * total nitrogen, *DO*  dissolved oxygen, *TP*  total phosphorous.

**Figure 2:**
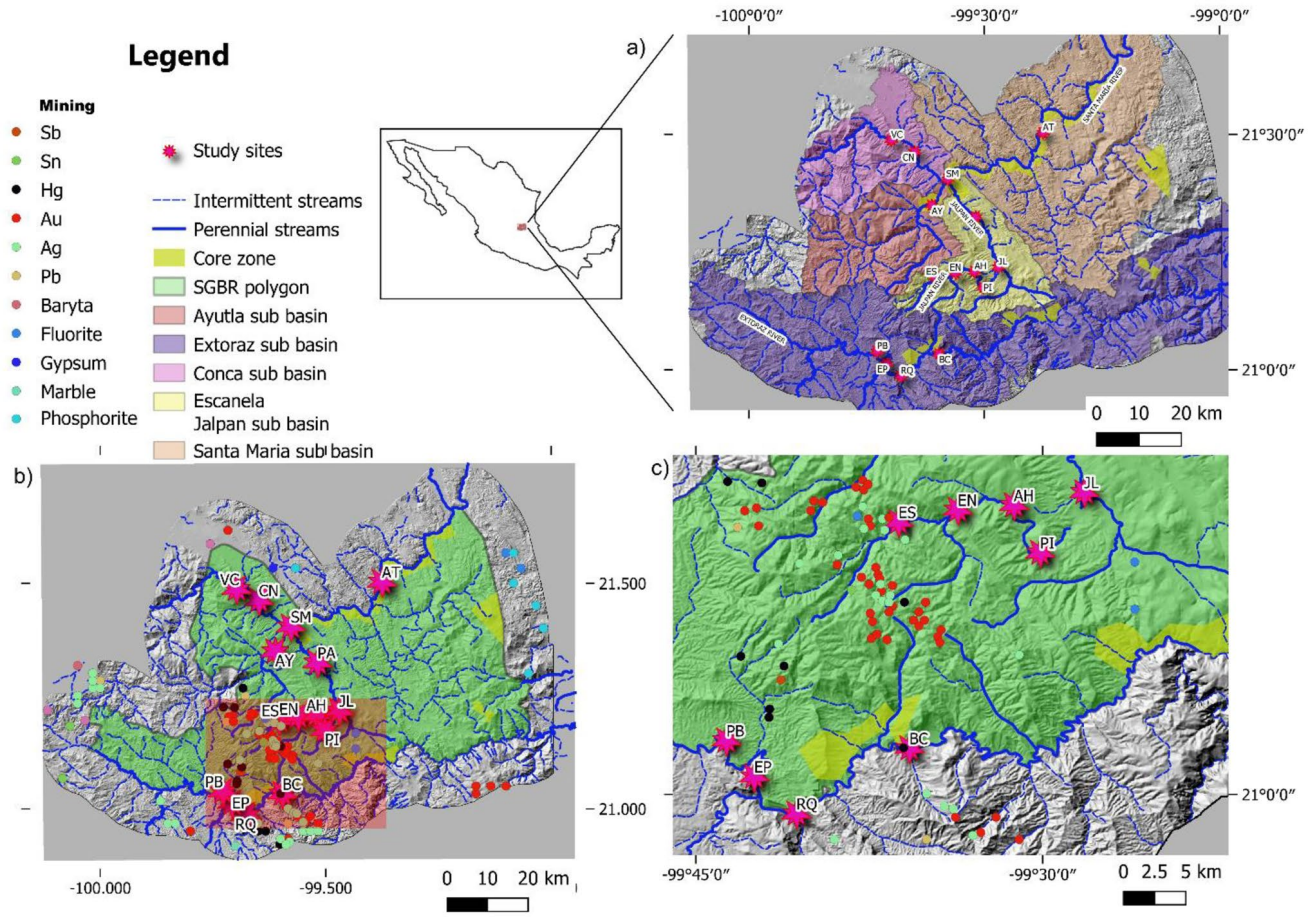
Scheme showing the streams and rivers of the Sierra Gorda Biosphere Reserve, **(a)** location of the 15 study sites, **(b)** mines located in the study area, and **(c)** zoom of the shaded area. The core zones of the reserve are areas under strict protection where any anthropogenic land use is forbidden. Acronyms identify the various sites as follows: *PB*  Peña Blanca, *EP*  El Paraíso, *RQ* Rancho Quemado, *BC* Bucareli, *ES* Escanela, *EN*  Escanelilla, *AH* Ahuacatlán, *PI* Pizquintla, *JL* Jalpan, *PA* Purísima de Arista, *VC* Vegas Cuatas, *CN* Concá, *AY* Ayutla, *SM* Santa María, *AT* Autopista 190. The map was generated using the vectorial layers freely available from National Institute of Statistics, Geography and Informatics (https://www.inegi.org.mx/temas/mapadigital), National Commission of Protected Natural Areas (http://sig.conanp.gob.mx/website/pagsig/info_shape.htm), and Mexican Geological Survey GEOINFOMEX (https://www.sgm.gob.mx/GeoInfoMexGobMx/). To build the map, all layers were processed with the open source software geographic information system QGIS 3.18 (QGIS is open source software available under the terms of the General Public License (GNU) meaning that its source code can be downloaded through tarballs or the git repository). Study sites points were downloaded from a hand-held GPS (Monterra®|Garmin) before being digitalized and uploaded as a shapefile.

Physical habitat quality showed contrasting values in some of the variables assessed, especially for vegetation protection, riparian cover, stream velocity, channel alteration, and maximum habitat quality score (Table 1). The worst conditions were observed in the Extoraz (sites PB, EP and RQ) and Escanela-Jalpan (sites AH, JL) Rivers, related to their proximity to human settlements, in contrast with higher scores in or near core zones (Escanela, Concá, Ayutla, and Santa María Rivers) (Fig. 2).

### Ordination of environmental factors

**Figure 3:**
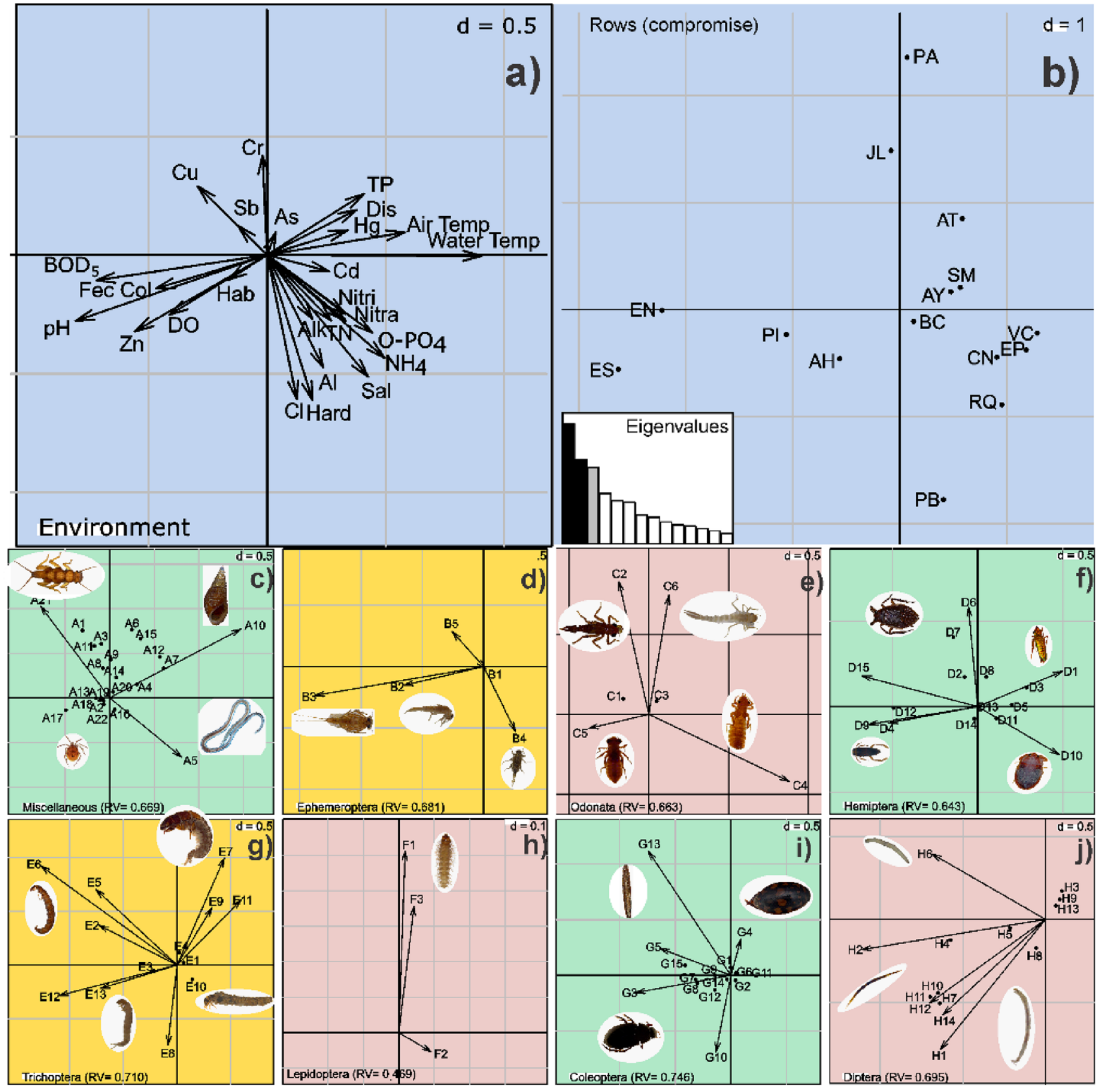
Results of the MCOA: **(a)** environmental factors resulting from the correlation analysis. Vectors indicate the magnitude of each factor over each taxa block; **(b)** ordered study sites; **(c–j)** blocks of contribution (vectors) of aquatic macroinvertebrate taxa (for easier visualization, separate biplots are shown for each taxonomic order). See Appendix for taxa codes. The blocks and MCOA analysis were generated using the ADE4 package (https://cran.r-project.org/web/packages/ade4/index.html; Dray, S. & Dufour, A.-B. 2007. The ade4 Package: Implementing the Duality Diagram for Ecologists) and vegan package (http://cran.r-project.org/;Oksanen, J., Kindt, R., Pierre, L., O’Hara, B., Simpson, G. L., Solymos, P., … Wagner, H. 2016. Vegan: Community Ecology Package, R package version 2.4–0). Images were edited using Inkscape, an open source software available at: https://inkscape.org.

The biplot of the co-inertia analysis shows the behavior of the selected environmental factors (Fig. 3a,b). Three groups of sites are differentiated: (1) Escanela-Jalpan River (sites ES, EN, AH, and PI) showed high BOD5 and fecal coliform levels resulting from inputs of municipal wastewater and high Zn concentrations; however, a basic pH, well-oxygenated waters, and the best habitat quality were predominant. (2) Extoraz River (sites PB, RQ, EP, and BC), Concá, and Santa María Rivers (sites VC and CN) were characterized by high hardness, alkalinity, salinity, total nitrogen, ammonia, nitrates, nitrites, and orthophosphates, all indicative of high mineralization and nutrient enrichment, in addition to high concentrations of heavy metals such as Al and Cd (suggesting erosion in the basin). (3) Ayutla and Santa María Rivers (sites AY, SM, AT, and PA) and one site in the Jalpan River (JL) showed high concentrations of total phosphorous, high temperature (air and water), high water discharge, and higher concentrations of metals such as Hg and As, which are indicative of pollution from urban areas and mining. The Jalpan River (site JL), particularly, had waters with high Cr, Sb, and Cu concentrations, demonstrating high pollution by materials transported from adjacent rivers (Fig. 2a,b).

### Relationship between environmental factors and patterns of macroinvertebrate assemblages

The BIOENV analysis showed a strong correlation of environmental parameters with macroinvertebrate assemblages in the SGBR. Variables that explained patterns in macroinvertebrate assemblages were total phosphorous, water temperature, air temperature, and Hg concentration (Spearman *rho* = 0.6). The other variables were not sufficiently correlated with the abundance of aquatic macroinvertebrates (see Supplementary Table S4).

The MCOA analysis revealed remarkable differences between the rivers studied as a result of the co-structure of richness and abundance of macroinvertebrate assemblages and environmental variables. The first two PCA axes accounted for 37% and 18% of total inertia. Most taxonomic groups (except for Lepidoptera) were highly correlated with particular environmental factors; RV values ranged from 0.64 to 0.80 (Fig. 3c–j). The three conditions of environmental factors and the rivers described above were related to particular macroinvertebrate assemblages. Most Hemiptera, Coleoptera, Diptera, and Trichoptera members (especially Corixidae, some Naucoridae, Polycentropodidae, Hydrophilidae, and Ceratopogonidae) showed a preference for well-oxygenated waters and the best habitat conditions (i.e., high vegetation density, heterogeneous substrates and vegetation types), in spite of the presence of fecal coliforms and high BOD_5_. Oligochaeta, Leptohyphidae, Gomphidae, and some Naucoridae were related to carbonates, nutrients, and higher Cd and Al levels. Most of the Odonata and macroinvertebrates in the Miscellaneous group, and some Trichoptera, Lepidoptera, and Diptera members, were related to high Cr, As, Hg, and TP concentrations.

## Discussion

We aimed to identify the distribution patterns of aquatic macroinvertebrate assemblages from sites with different metal concentrations in water, significantly influenced by mining operations located in the upper reaches of the rivers. A set of environmental factors was examined to explore their correlations with biological data. To this end, we used multivariate analysis MCOA (Dolédec and Chessel^31^), which helped elucidate the main environmental factors influencing aquatic macroinvertebrate assemblages. The MCOA combines separate ordinations into a single analysis based on the cross-covariance matrix^32^. We first identified and removed the environmental variables that were redundant, to improve the analysis. Thus, any potential biases in our results were avoided by selecting a set of variables of similar prediction efficacy^33,34^.

This study highlights the high diversity of aquatic macroinvertebrates inhabiting the SGBR (see “Appendix”). High diversity values were found mainly in rivers located in the central and northwestern locations of the SGBR (Escanela-Jalpan and Santa María Rivers). In general, these rivers have suitable conditions for macroinvertebrates, i.e., good habitat quality and differences in the adjacent vegetation (ranging from pine and deciduous forests to xeric shrubland^35^) between the river reaches. However, the wastewater treatment facility (WWTF) located between sites EN and AH on the Escanela River is associated with a lower diversity (^0^*D*, ^1^*D*, and Shannon entropy *H’*) that can be attributed to unsuitable conditions derived from habitat deterioration and unfavorable water quality affecting the establishment of various macroinvertebrates. According to Vinson^36^, factors including habitat fragmentation, changes in water discharge, temperature, and poor water quality are strong environmental disturbances for the establishment of aquatic macroinvertebrates in rivers. Water discharge is reduced (even interrupted, causing fragmentation) downstream of the WWTF, and, therefore, the local macroinvertebrate assemblages showed a marked reduction in taxa richness (^0^*D*) and other diversity measures (^1^*D* and Shannon entropy *H’*). Torres-Olvera et al.^37^ demonstrated poor water quality in the Extoraz River using the Index of Biological Integrity based on Macroinvertebrate Assemblages (IIBAMA). These studies are consistent with our results showing low richness (^0^*D*) and moderate diversity (^1^*D* and ^2^*D*). Different results were observed in the Concá River, where good quality was determined by IIBAMA. Contrasting with Torres-Olvera et al.^37^, we found low diversity (^0^*D* and ^1^*D*) and moderate exponential Shannon’s diversity (^2^*D*), which indicate low abundance of several taxa in the system.

Carabias-Lillo et al.^38^ mentioned the high diversity of the vegetation in some portions of the SGRB, describing the dominance of *Quercus*, *Pinus*, and deciduous forests (Escanela-Jalpan, Santa María, Concá, and Ayutla Rivers), contrasting with the xeric shrubland in Extoraz River. Therefore, macroinvertebrate diversity is likely associated with several variables. Among them, the adjacent vegetation may strongly influence taxonomic diversity of aquatic macroinvertebrates; in addition, other environmental variables (geophysical landscape, land use, vegetation cover, and site habitat) influence diversity in macroinvertebrate assemblages, as stated by Macedo et al.^39^. As mentioned above, the Escanela-Jalpan and Santa María basins are dominated by pine forest and tropical deciduous forest^38^, respectively, and both basins reached high macroinvertebrate diversity values. Studies from Callisto et al.^40^ have highlighted the importance of tree leaves for macroinvertebrates in relation to habitat availability, as well as the high turnover between habitats (increased beta diversity), a condition that may favor the exchange of biota across rivers. Additionally, those rivers provide the best conditions in terms of habitat suitability for living organisms. In contrast, the Extoraz basin is largely covered by xeric vegetation (low vegetation cover), according to Gutiérrez-Yurrita et al.^35^. The Extoraz River showed the lowest taxa diversity (^0^*D* and ^1^*D*) and moderate dominance (^2^*D*), which may be influenced by contrasting degrees of vegetation protection in different areas of the SGBR. Semi-arid environments, such as the Extoraz River, offer poor-quality shelter for macroinvertebrates because of naturally reduced (spatial and temporal) riverbank and riparian vegetation^41^. Hence, low habitat quality is frequently observed (and extremely low quality in areas under intensive mining). Despite the relatively low diversity and poor habitat conditions in the Extoraz River, we identified 32 different taxa (mostly at family level). By comparison, López-López et al.^42^ found 40 macroinvertebrate families in the Salado River, which flows across another Mexican PNA with similar vegetation, using the same sampling methods. However, in the Salado River, studies were conducted in two sampling sites, whereas our study was conducted in four study sites (Extoraz River). Other similar studies carried out by Ruiz-Picos et al.^23^ in Central Mexico recorded 66 taxa (63 at family level) in duplicated samples across a network of two rivers and 14 study sites in tropical rivers. Several authors have pointed out the importance of riparian vegetation and mature trees to provide protection and habitats for the establishment of fish and macroinvertebrates^43,44^; these same conditions may increase the diversity of macroinvertebrates in the SGBR. Such is the case of the Escanela-Jalpan and Santa María Rivers where large mature trees are abundant, in agreement with Gutiérrez-Yurrita et al.^35^, contrasting with the Extoraz River.

The Hill diversity numbers (^1^*D* and ^2^*D*), showed an increasing trend downstream reaching as far as the Santa María River, which flows across the lower portions of the basin. According to Vannote^45^ and Malmqvist and Hoffsten^46^, aquatic diversity tends to be higher in downstream reaches because of the contribution of tributaries flowing into the mainstream along its gradient and habitat heterogeneity. We found marked differences between Shannon’s diversity index (*H*’) values and Hill numbers. Shannon’s index was not as sensitive to minor differences in diversity between and within the rivers studied as the Hill numbers were. As Shannon’s index is based on the amount of information provided by the identity of an individual chosen at random, rare or uncommon species are not expected. Jost et al.^47^ mentioned that linear diversity calculations (such as Shannon’s index) are not entirely suitable to determine diversity. For this reason, we calculated Hill numbers to estimate the “effective number of species”. The corrected diversity values provided by Hill numbers showed a pattern of increasing diversity downstream, which was most evident in the Concá and Ayutla Rivers. In most cases, assemblage diversity is determined by a set of environmental conditions^48^.

We assumed that the contrasting diversity (dominance and taxa richness) between the Extoraz and Escanela Rivers was determined by a set of environmental factors and the tolerance of some taxa to the presence of heavy metals. As stated above, our results coincide with those of Jerves-Cobo et al.^49^, who studied the impact of sewage on aquatic macroinvertebrate assemblages along an altitudinal gradient in Cuenca, Ecuador. Those authors identified a higher influence of DO and DBO_5_ than nutrients as drivers of biological water quality according to biomonitoring tools^49^; however, they did not find impacts from mining operations in the basin. We found high concentrations of heavy metals (particularly Cd and Hg) in the sites studied; in some cases, those concentrations exceeded the water quality criteria for aquatic life established by the US EPA^50^. Heavy metals exert adverse biological effects that are magnified in upper trophic levels; heavy metal precipitates hamper growth in periphyton due to nutrient reduction and photosynthesis inhibition^51^, affecting the structure of macroinvertebrates that feed on it and other sources in the substrata. Therefore, our results show the effects of mining activities in the SGBR, as it is the most common human activity in the upper reaches of the rivers flowing through the SGBR^52^. According to Robles et al.^53^, cinnabar (HgS) attains high concentrations due to illegal mining. The SGRB has been historically referred to as a well-mineralized place, mainly by cinnabar (HgS), which is the most common ore in the SGBR and the primary mercury-containing mineral. Mercury production in the area has been high in the past century through illegal mining^54,55^. Erosion processes may further lead to high mercury concentration in water (peak values of 0.049 mg L^−1^ in the present study). As per the water quality criteria for aquatic life established by the US EPA^50^, the maximum mercury concentration in freshwater is 0.0014 mg L^−1^. However, the concentrations recorded in SGBR water were twice as high. Thus, the SGBR is at imminent risk of heavy metal pollution, particularly by mercury, as Hernández-Silva et al.^54^ have reported for maize crops in the SGBR. Cadmium is another metal whose levels in SGBR rivers are twice the upper limit (0.00025 mg L^−1^) set by the US EPA^50^. Other heavy metals and metalloids (Al, As, Cr, Cu, and Sb) measured in this study did not exceed the limits compatible with aquatic life. Nevertheless, their concentration may increase in the future if erosion proceeds at the current rate. Our analyses revealed that a well-mineralized environment prevails in the rivers of the SGBR. High CaCO3 concentrations, evidenced by alkalinity and hardness (Table 1), are indicative of a particular geological stratum. Moreover, the soil types Regosol, Vertisol, Litosol, Rendzina, and Cambisol, were identified by Carrillo-Martínez and Suter-Cargneluti^56^. These findings suggest that long-term erosion of soluble rocks naturally occurs in the area^52^.

We found several nitrogen compounds directly linked to current human activities in the area, such as urbanization and agriculture. Mostly rural settlements occur in the SGBR (population density of 25 inhabitants per km^2^) and sewage treatment is scarce, with only three municipal wastewater treatment plants currently operating. However, wastewater discharges from the larger villages (as in the Escanela-Jalpan basin) are starting to affect the nearby rivers. Total nitrogen and total phosphorus reached concentrations above 4 mg L^−1^ and 0.7 mg L^−1^, respectively, usually associated with extensive deforestation and agriculture in impacted basins^57,58^. Khatri and Tyagi^59^ have discussed the impacts of rural and urban sewage, where farming and runoff from human settlements are the primary factors increasing the input of coliforms and organic compounds into nearby stream water. We identified villages such as Ahuacatlan (AH) and Jalpan (JL), located in the Escanela basin, as well as river modifications such as the reservoir on the Jalpan River, that may cause adverse effects on the ecosystem^57,60^.

The present study identified a set of explanatory variables that influence the abundance of macroinvertebrate assemblages in the SGBR. Temperature (air and water) is a key variable influencing multiple aspects of aquatic ecosystems and their biota. Consequently, it was selected as a variable with high influence on macroinvertebrates according to BIOENV. The upper temperature limit is one of the key factors that determines the relative sensitivity of organisms, showing important survival thresholds. Several heat-tolerant organisms may be useful as bioindicators due to their unique survival strategies (behavior, feeding, growth, metabolic rates, emergence, and fecundity) that make them thrive^61^. Meanwhile, rising phosphorous concentrations may cause significant damages to freshwater organisms. Struijs et al.^62^ have stressed the importance of nutrient enrichment (> 0.3 mg L^−1^) that may potentially lead to the disappearance of some macroinvertebrate genera; a concentration of 3.5 mg L^−1^ may cause the elimination of half of the macroinvertebrate genera in the water column. Mercury was detected as another explanatory variable by BIOENV; however, a reduced number of organisms are able to survive in high Hg concentrations. Despite well-mineralized and carbonated waters in SGBR rivers, precipitates of metals and uptake of methylmercury may lead to adverse effects on all trophic levels^63^. Metal precipitates may reduce periphyton, affecting trophic chains and, consequently, increasing the dominance of tolerant macroinvertebrates^51^.

The MCOA analysis revealed a significant relationship between key environmental factors and macroinvertebrate families pooled into groups in a spatial dimension. Corkum^64^ highlighted that multivariate analyses are very useful tools to assess macroinvertebrate assemblages. The MCOA yielded RV values for each taxonomic group that were generally high (0.46 to 0.74). Dalu et al.^65^ examined approximately 5000 specimens in short-term studies and found RV values that rarely reached a maximum of 0.30. In spite of their high number of sampled sites (84) in a Neotropical region, only 57 taxa were found in the whole study. We examined more than 70,000 specimens sorted into 93 taxa, which made it possible to achieve high correlation values between environmental factors and macroinvertebrate assemblages, reflecting the high sampling effort in only 15 sampling sites. This is particularly true relative to broader studies by Torres-Olvera et al.^37^, who sampled 33 sites (with a sampling effort of 30 minutes with no replicate samples per site) and collected 10 723 specimens from 86 families in the same basin and other adjacent basins in the SGBR. The above suggests the omission of many taxa (that we collected), indicating the importance of sufficient sampling effort. The abundance of macroinvertebrates is frequently overlooked as a result of subsampling or reduced sample size^29^; however, we realized the importance of the number of individuals to detect biological patterns. Often, the combination of various biomonitoring approaches warrants predictive modelling, but requires major efforts in terms of sampling design and expertise; however, taxonomic knowledge of macroinvertebrate assemblages is far from being complete in Mexico and Latin America as a whole^66^.

Overall, we identified three different conditions for aquatic life in the SGBR: (a) water with high concentrations of heavy metals derived from mining activities, (b) water with high nutrient concentrations from villages and primary activities carried out in the area, and (c) well-oxygenated water with high coliform numbers (Fig. 3a). Using the MCOA, we were able to correlate particular macroinvertebrate families with each of these three water quality conditions. Thus, we found that aquatic macroinvertebrate assemblages showed patterns related to environmental conditions. Several families clustered in the miscellaneous group of aquatic macroinvertebrates were related to heavy metal concentrations; the vectors representing most of them appear on the upper part of the PCA biplot (Fig. 3b–h) and show their association with heavy metals. Studies carried out in a mining area in Cajamarca, Peru^67^, indicated the impact of sewage on macroinvertebrate assemblages, but with no apparent influence of mining pollution, according to the biomonitoring procedures used (Biological Monitoring Working Party Colombia, which is focused on organic pollution). Consequently, these biomonitoring tools were seemingly unsuitable for identifying mining impact on those assemblages. In contrast, studies carried out in Australia by Wright et al.^28^ and in Portugal by Gerdhart et al.^68^ have reported ecological impairment in rivers affected by mining and industrial operations on aquatic macroinvertebrates identified at the family level (with Ephemeroptera, Plecoptera, and Trichoptera as the most sensitive taxa). In our study, we highlight the taxonomic groups related to the presence of metals (from artisanal mining). Groups such as Plecoptera, mollusks, and worms showed a close relationship with municipal wastes and high concentrations of metals (Cu, Cr, Sb, As, and Hg). In a study in high-mountain streams of the Gangqu River, China, Qu et al.^69^ found an inverse relationship between heavy metal concentration and diversity of macroinvertebrate assemblages, particularly affecting Ephemeroptera, Plecoptera, and Trichoptera taxa (EPT). Other studies have shown the association between aluminum concentration in the water column and the presence of Perlidae such as the genus *Anacroneuria*^25,70^; however, we found a different response in the SGBR. The family Perlidae was sensitive to high aluminum and nutrient content in water, regardless of the concentration of other heavy metals, including mercury. Other groups such as mollusks, worms, and flatworms were highly correlated with high concentrations of heavy metals. Ankley^71^ and Croteau et al.^72^ found bioaccumulation of Cd, Cu, Pb, Ni, and Zn from sediments in several mollusks, Perlidae, and worms, confirming that these taxa are tolerant to high concentrations of heavy metals. We found Leptophlebiidae and Psephenidae living in water with high Cd, Hg, and Al levels. High Cu, Cr, Cd, and As concentrations have been related to high abundances of some Ephemeropterans (Leptophlebiidae), and Coleopterans (Psephenidae)^68,73^. Our findings further indicate that these taxa are tolerant to heavy metals. Some Odonata (Coenagrionidae and Platystictidae) were also related to high concentrations of heavy metals. Studies by Corbi et al.^74^ and Michailova et al.^27^ suggest that some Odonata taxa are tolerant to heavy metals in water. Our results are consistent with those studies, as we found a high correlation between the presence of Odonata (Coenagrionidae and Platystictidae) and high Cd and Al levels. We found similar responses in some Hemiptera and Trichoptera. However, Hemiptera are air-breathing, many skate on the water surface, and, eventually, leave the aquatic environment^75^, traits that, taken together, allow them to thrive in polluted water. Trichoptera are considered less tolerant to pollution than non-EPT insects^76^. Nonetheless, some Trichoptera families, e.g., Hydropsychidae and Philopotamidae, have been recognized as tolerant to heavy metals in water^28,29,69^. We found several families of Trichoptera, particularly Hydropsychidae, Philopotamidae, and Hydrobiosidae, that were correlated with As and Hg levels. In contrast, the presence of Lepidoptera was insufficient to relate it to pollution by heavy metals; only the family Crambidae showed tolerance to heavy metals. Ephemeroptera such as Baetidae and Heptageniidae showed a high affinity for well-oxygenated water, while Leptophlebiidae displayed high tolerance to heavy metals (As and Hg) (Fig. 3c). Several authors mention that the Ephemeroptera life cycle depends on water temperature in running water^77,78^, and that they prefer cool well-oxygenated water. As Jacobsen et al.^79,80^ found in previous studies, this condition is generally true for Ephemeroptera, Plecoptera, and Trichoptera. We observed the same pattern in some Ephemeroptera, but not in all of them. Heptageniidae and Baetidae are highly sensitive to the content of heavy metals in water, but Baetidae tolerates water rich in coliforms. Those responses have also been observed in several studies under laboratory conditions; for example, Clements^26^ and Courtney and Clements^81^ demonstrated that Heptageniidae is highly sensitive to heavy metals such as Zn, Cd, and Cu. In general, Ephemeroptera are sensitive to heavy metals; however, Baetidae and Leptophlebiidae are the most diverse families of mayflies, and their responses are equally diverse^82^. Baetidae (genus *Baetis*) is a genus well-known for tolerating heavily polluted water^77^. Although Baetids and Heptageniids tolerate coliform bacteria from sewage, they are potentially suitable indicators of water quality, particularly as regards the impact of mining activity in the SGBR. The families Lumbriculidae, Leptohyphidae, Gomphidae, and Naucoridae were closely related to nutrient-enriched water; also, Oligochaeta includes nutrient-tolerant taxa; Ristau et al.^83^ demonstrated changes in their density driven by peaks in phosphorous concentration. We found the same pattern with Leptohyphidae, which showed tolerance to water containing high nutrient concentrations.

We found high metal concentrations in SGBR rivers; however, a high diversity of aquatic macroinvertebrates was also detected. The geological strata of the reserve (mainly limestone shale and dolomite limestone) contribute to high water hardness and alkalinity. Both factors reduce the bioavailability and toxic effect of metals on aquatic life^50^. Thus, a buffer effect is likely to occur, given the high concentrations of carbonates (CO3^−^ and HCO3^−^) in the study area, which form soluble or insoluble complexes or precipitates^84^. High water alkalinity reduces the toxicity of metal ions either by active surface competition for binding sites in tissues^85^ or by reducing their concentration through the formation of insoluble precipitates. Furthermore, it is likely that Ca^2+^ and Mg^2+^ from carbonates may compete with other divalent metal ions for binding sites in organisms^86^. Carbonate functions as a blocker to the entry of metals into organisms; hence, high water alkalinity and hardness in SGBR rivers are probably acting synergistically to decrease the availability and toxicity of heavy metals, thus protecting the aquatic biota to some degree. However, this effect was less evident in the Extoraz River, which has xeric vegetation^35^ , the highest number of mines in the upper reaches, and the highest salinity; taken together, these factors contribute to the lower diversity of aquatic macroinvertebrates in this river. The results of this study using the MCOA revealed diverse macroinvertebrate assemblages that can be ranked along a stress gradient (heavy metals concentration), ranging from families that are highly sensitive to others that are tolerant to high metal concentrations. This allowed us to identify taxa that seem suitable for use as indicators of the ecological health of rivers affected by mining activities. Our study is one of the first efforts in Latin America to characterize the response of macroinvertebrates in support of their use as biomonitoring tools in areas influenced by mining operations.

## Methods

### Study area

The SGBR is one of the most important PNAs in Mexico, with a surface area of 3800 km^2^^87^, located in the Central Mexican Plateau, between the Nearctic and Neotropical biogeographical regions^38^. This PNA is located in a mountain range called “Sierra Gorda”, with two major rivers: the Santa María River in the northeast and the Extoraz River in the south. Both are tributaries of the Panuco River (which flows into the Gulf of Mexico). Data were collected at 15 sites across the SGBR along the Extoraz and Santa María Rivers and their tributaries (Concá, Ayutla, and Escanela-Jalpan Rivers) (Fig. 2). A systematic selection of 15 study sites was conducted to include all streams flowing across the SGBR, with some accessibility and security constraints. We also selected some sampling sites at upper reaches that receive no sewage and other sites downstream of sewage sources (i.e., WWTF). Two main villages in the SGBR (Ahuacatlán and Jalpan) are located along the Escanela-Jalpan River; a WWTF is located on the periphery of Ahuacatlan; the stream then flows into a reservoir before joining the Santa María River. Several small towns with some 93 000 inhabitants are located in the SGBR; small parts of the area are used as cropland^88^. The main geological strata in the SGBR are limestone^21^, shale, and dolomite. Sierra Gorda is home to one of the most diverse vegetation types among Mexican protected natural areas. The main vegetation types in the SGBR are pine forest (Escanela River), tropical rainforest, oak forest, deciduous tropical forest (Santa María River), and xeric shrubland (Extoraz River). The local climate ranges from semi-warm to warm and sub-humid to semi-dry with an annual mean temperature mostly above 18 °C. Precipitation averages 313 mm in the dry season and 883 mm in the summer rainy season^89^. Currently, 140 mines are located in Sierra Gorda with 83 in the SGBR, all of them in the upper reaches of the five sub-basins that converge into the mainstreams (Extoraz, Escanela, Jalpan, Concá, Ayutla, and Santa María Rivers). Mining operations affect the entire basin downstream of the mining area where wastes are dumped (Fig. 2b,c). Most mines produce Au (46%), Ag (27%), Hg (8%), Pb (5%), and Sn (2%), as well as non-mineral materials (Sb, barite, fluorite, phosphorite, marble, and gypsum, 12%). The numbers of mines are as follows: Extoraz, 46; Escanela Jalpan, 40; Ayutla, 28; Concá, 1; and Santa María, 2. In most cases, mining operations are artisanal^21^.

### Environmental factors

The geographic coordinates and elevation (in m asl) of each sampling site were recorded with a GPS Garmin^®^ device. At each sampling site, the environmental variables recorded were water and air temperature (°C), dissolved oxygen (mg L^−1^), pH, turbidity (NTU), conductivity (mS cm^−1^), salinity (PSU) (using a Quanta^®^ probe). Water samples were collected on two occasions in the dry (February 2017 and January 2018) and rainy (July and October 2017) seasons. In all cases, seasonal averages were calculated for each sampling site. The concentrations of nutrients, major ions, and other physicochemical factors (Supplementary Table S5) were measured using HACH^90^ and APHA^91^ procedures. Heavy metal and metalloid concentrations were measured after microwave acid digestion (Anton-Paar® Multiwave Go) (EPA 3015A) using the methods recommended by the relevant Mexican regulations^92^. The digested samples were analyzed using Inductively Coupled Plasma Optical Emission Spectrometry (ICP OES) (Perkin Elmer® Optima 4300DV). Metal concentrations below the detection limit were arbitrarily assigned a value of one-half of the respective detection limit^93^. In each 100-m reach, physical habitat quality—a summary description of the variety of habitats and their features—was evaluated by a visual-based habitat assessment (VBHA). Scores were estimated from habitat surveys, assigning a score to physical features including stream morphology, substratum, riparian coverage, and status of the floodplain, using an ordinal categorical scale^94,95^. VBHA scores were then used to compute a habitat score following the procedures described by Barbour et al.^94^; physical habitat scores ranged from 0 to 20, with higher values indicating more heterogeneous habitats. River discharge was estimated according to Michaud and Wierenga^96^. For each environmental factor and habitat structure, the mean of four replicate measurements was calculated. Data on environmental factors and habitat variables (water and habitat quality) were tested for collinearity using the Spearman’s rank correlation coefficient to exclude redundant variables from further analysis.

### Macroinvertebrates

Aquatic macroinvertebrates were sampled at each study site in February (dry season) and July (rainy season) 2017, using multi-habitat methods (Barbour et al.^94^, US National Rivers Assessment^95^, AQEM^97^. Four 5-minute subsamples were collected at each site, then pooled immediately into a 20-minute sample, including stream detritus collected in the net, and stored in a container for each sampling site. Two subsamples were collected in riffle sections using a kick net, and the other two in the most dominant habitat using a scoop net; both nets had a 500 µm mesh size. Specimens were preserved in 70% alcohol for subsequent cleaning, sorting, and identification in the laboratory. These specimens were identified using specialized literature for North American and Neotropical areas (Merrit and Cummins^98^, Thorp and Covich^99^, Bueno-Soria^100^, Springer et al.^101^, and Hamada et al.^102^). Taxa were primarily determined to family level. Taxa from the two seasons were pooled together into a single list to compute mean values for each study site.

### Data analyses

The diversity of macroinvertebrates at each study site was evaluated using four biodiversity indices. The Hill diversity numbers or “true diversities” and the Shannon entropy index (*H’*) were calculated using the method described by Jost et al.^103^. In this method, assemblage diversity measures are converted into “effective number of species” and three Hill diversity numbers of different orders (*q*) can be assessed. The parameter *q* controls the sensitivity to common and rare species. When *q* = *0*, abundance values are raised to the power of 0, and rare and abundant species have the same weight; thus, the Hill diversity number of order 0 (^0^*D*) represents taxa richness. When *q* = *1*, abundance values are raised to the power of 1, and the Hill diversity number of order 1 (^1^*D*) represents the exponential of Shannon’s diversity index. When *q* = *2*, abundance values are raised to the power of 2, thus increasing the weight of dominant species, and the Hill diversity number of order 2 (^2^*D*) represents the inverse of the Gini-Simpson’s dominance index^103^. A distinctive advantage of this approach is that all diversity numbers can be interpreted as effective numbers of species. Diversity profiles for each river were summarized as heatmaps. Diversity indices were computed with the package iNEXT in R 3.1.0. The data were first tested for normality and homoscedasticity, and then a one-way ANOVA was used to test for differences in diversity indices between rivers.

To obtain explanatory variables of relationship between environmental factors and abundance of macroinvertebrate assemblages, a BIOENV correlation analysis was performed with the package Vegan in R 3.1.0. Hence, we investigated how environmental factors were correlated with macroinvertebrates assemblages. Then, the relationship between environmental factors and aquatic macroinvertebrates at the different study sites was examined. First, we constructed an environmental data matrix containing the mean values of each of the 25 environmental factors recorded at the 15 study sites and the two sampling seasons. Macroinvertebrate abundance data were log-transformed [ln (*x*+1)] to reduce the effect of dominant taxa, and then a matrix of species by sites was built. Afterward, these two matrices were subjected to a multiple co-inertia analysis (MCOA) (999 permutations) following Dolédec and Chessel^31^. We performed a multi-table STATIS analysis to identify the relationship between taxa (orders and a group of miscellaneous families) and environmental factors spatially, according to Lavit et al.^104^. Finally, to evaluate the significance of the relationships between taxa and environmental factors, we calculated RV (*rho* values). These analyses were carried out using the packages ADE4 and Vegan 2.0.10 in R 3.1.0. Ordination biplots showing the relationship between environmental factors and taxa were constructed; separate biplots in blocks for each order (including its respective families) were constructed for easier visualization.

### Supplementary Information

Supplementary Tables.

## Appendix

**Table:** Taxa codes of sampled macroinvertebrates in the hydrological systems of BRSG and projected in Fig. 3.

Taxa	Code	Taxa	Code	Taxa	Code
Planariidae	A1	Libellulidae	C5	Noctuidae	F2
Goordiidae	A2	Platystictidae	C6	Pyralidae	F3
Hirudinidae	A3	Aphididae	D1	Chrysomelidae	G1
Haplotaxidae	A4	Belostomatidae	D2	Curculionidae	G2
Lumbriculidae	A5	Cicadelidae	D3	Dryopidae	G3
Corbiculidae	A6	Corixidae	D4	Dytiscidae	G4
Pisidiidae	A7	Gelastocoridae	D5	Elmidae	G5
Planorbidae	A8	Gerridae	D6	Georyssidae	G6
Pleuroceridae	A9	Hebridae	D7	Gyrinidae	G7
Thiaridae	A10	Hydrometridae	D8	Haliplidae	G8
Ancylidae	A11	Macroveliidae	D9	Hydraenidae	G9
Physidae	A12	Naucoridae	D10	Hydrophilidae	G10
Asellidae	A13	Nepidae	D11	Lampyridae	G11
Atyidae	A14	Notonectidae	D12	Lutrochidae	G12
Ostracoda	A15	Ochteridae	D13	Psephenidae	G13
Collembola	A16	Saldidae	D14	Scirtidae	G14
Trombidiformes	A17	Veliidae	D15	Staphylinidae	G15
Anthicidae	A18	Brachycentridae	E1	Ceratopogonidae	H1
Blaberidae	A19	Calamoceratidae	E2	Chironomidae	H2
Tridactylidae	A20	Ecnomidae	E3	Culicidae	H3
Perlidae	A21	Glossosomatidae	E4	Dixidae	H4
Corydalidae	A22	Helicopsychidae	E5	Dolichopodidae	H5
Acanthametropodidae	B1	Hydrobiosidae	E6	Empididae	H6
Baetidae	B2	Hydropsychidae	E7	Ephydridae	H7
Heptageniidae	B3	Hydroptilidae	E8	Muscidae	H8
Leptohyphidae	B4	Leptoceridae	E9	Phoridae	H9
Leptophlebiidae	B5	Odontoceridae	E10	Psychodidae	H10
Calopterygidae	C1	Philopotamidae	E11	Simuliidae	H11
Coenagrionidae	C2	Polycentropodidae	E12	Stratiomyidae	H12
Corduliidae	C3	Xiphocentronidae	E13	Tabanidae	H13
Gomphidae	C4	Crambidae	F1	Tipulidae	H14

## References

[1] Marqués MJ, Martínez-Conde E, Rovira JV, Ordóñez S (2001). Heavy metals pollution of aquatic ecosystems in the vicinity of a recently closed underground lead-zinc mine (Basque Country, Spain). Environ. Geol..

[2] Bud I, Duma S, Denuţ I, Taşcu I (2007). Water pollution due to mining activity. Causes and consequences Wasserverunreinigung aufgrund von Bergbauaktivitäten. Ursachen und Konsequenzen. BHM Berg- Hüttenmännische Monatsh..

[3] Ugya Y (2017). Assessment of ambient air quality resulting from anthropogenic emissions. Am. J. Prev. Med. Public Health.

[4] Dore E (2000). Environment and society: Long-term trends in Latin American mining. Environ. Hist. Camb..

[5] Zhou Q (2020). Total concentrations and sources of heavy metal pollution in global river and lake water bodies from 1972 to 2017. Glob. Ecol. Conserv..

[6] Graesser J, Aide TM, Grau HR, Ramankutty N (2015). Cropland/pastureland dynamics and the slowdown of deforestation in Latin America. Environ. Res. Lett..

[7] Ramírez A, Pringle CM, Wantzen KM (2008). Tropical stream conservation. Trop. Stream Ecol..

[8] Uriarte M, Yackulic CB, Lim Y, Arce-Nazario JA (2011). Influence of land use on water quality in a tropical landscape: A multi-scale analysis. Landsc. Ecol..

[9] White M, Barquera S (2020). Mexico adopts food warning labels, why now?. Health Syst. Reform.

[10] Koleff, P. *et al.* Biodiversity in Mexico: State of knowledge. in *Global Biodiversity*. 285–337. 10.1201/9780429433634-8. (Apple Academic Press, 2018).

[11] Armendáriz-Villegas EJ (2015). Metal mining and natural protected areas in Mexico: Geographic overlaps and environmental implications. Environ. Sci. Policy.

[12] Montoya-Lopera P (2020). New geological, geochronological and geochemical characterization of the San Dimas mineral system: Evidence for a telescoped Eocene-Oligocene Ag/Au deposit in the Sierra Madre Occidental, Mexico. Ore Geol. Rev..

[13] LeuraVicencio AK, CarrizalesYañez L, RazoSoto I (2017). Mercury pollution assessment of mining wastes and soils from former silver amalgamation area in North-Central Mexico. Rev. Int. Contam. Ambient..

[14] Veiga, M. M. *Introducing New Technologies for Abatement of Global Mercury Pollution in Latin America*. *United Nations Industrial Development Organization (UNIDO), University of British Columbia (UBC), Center of Mineral Technology (CETEM)* (UNIDO, UBC, CETEM, 1997).

[15] Camacho A (2016). Mercury mining in Mexico: I. Community engagement to improve health outcomes from artisanal mining. Ann. Glob. Health.

[16] IUCN. *Benefits Beyond Boundaries: Proceedings of the Vth IUCN World Parks Congress : Durban, South Africa. 8–17 September 2003*. (Iucn, 2005).

[17] González SO, Almeida CA, Calderón M, Mallea MA, González P (2014). Assessment of the water self-purification capacity on a river affected by organic pollution: Application of chemometrics in spatial and temporal variations. Environ. Sci. Pollut. Res..

[18] Rico-Sánchez AE (2020). Biological diversity in protected areas: Not yet known but already threatened. Glob. Ecol. Conserv..

[19] Harvey CA (2008). Integrating agricultural landscapes with biodiversity conservation in the Mesoamerican hotspot. Conserv. Biol..

[20] Messerli B, Grosjean M, Vuille M (1997). Water availability, protected areas, and natural resources in the Andean desert altiplano. Mt. Res. Dev..

[21] Servicio Geológico Mexicano. Conoce GeoInfoMex en 3D. https://www.gob.mx/sgm/articulos/conoce-el-sistema-de-consulta-de-informacion-geocientifica-geoinfomex?idiom=es. Accessed 18 Feb 2021. (2019).

[22] Resh VH (2008). Which group is best? Attributes of different biological assemblages used in freshwater biomonitoring programs. Environ. Monit. Assess..

[23] Ruiz-Picos, R. A., Sedeño-Díaz, J. E. & López-López, E. Calibrating and validating the biomonitoring working party (BMWP) index for the bioassessment of water quality in neotropical streams. in *Water Quality* (InTech, 2017).

[24] Oertel, N. & Salánki, J. Biomonitoring and bioindicators in aquatic ecosystems. in *Modern Trends in Applied Aquatic Ecology*. 219–246. 10.1007/978-1-4615-0221-0_10. (Springer, 2011).

[25] Goodyear KL, McNeill S (1999). Bioaccumulation of heavy metals by aquatic macro-invertebrates of different feeding guilds: A review. Sci. Total Environ..

[26] Clements WH (2004). Small-scale experiments support causal relationships between metal contamination and macroinvertebrate community responses. Ecol. Appl..

[27] Michailova P, Warchałowska-Śliwa E, Szarek-Gwiazda E, Kownacki A (2012). Does biodiversity of macroinvertebrates and genome response of *Chironomidae larvae* (Diptera) reflect heavy metal pollution in a small pond?. Environ. Monit. Assess..

[28] Wright IA, Ryan MM (2016). Impact of mining and industrial pollution on stream macroinvertebrates: Importance of taxonomic resolution, water geochemistry and EPT indices for impact detection. Hydrobiologia.

[29] Wright IA, Burgin S (2009). Comparison of sewage and coal-mine wastes on stream macroinvertebrates within an otherwise clean upland catchment, Southeastern Australia. Water Air Soil Pollut..

[30] Batty LC (2005). The potential importance of mine sites for biodiversity. Mine Water Environ..

[31] Dolédec S, Chessel D (1994). Co-inertia analysis: An alternative method for studying species–environment relationships. Freshw. Biol..

[32] Thioulouse, J. *et al. Multivariate Analysis of Ecological Data with ade4*. *Multivariate Analysis of Ecological Data with ade4*. 10.1007/978-1-4939-8850-1. (Springer, 2018).

[33] Dodds WK, Clements WH, Gido K, Hilderbrand RH, King RS (2010). Thresholds, breakpoints, and nonlinearity in freshwaters as related to management. J. N. Am. Benthol. Soc..

[34] Sundermann A, Gerhardt M, Kappes H, Haase P (2013). Stressor prioritisation in riverine ecosystems: Which environmental factors shape benthic invertebrate assemblage metrics?. Ecol. Indic..

[35] Gutiérrez-Yurrita PJ, García-Serrano LA, Plata MR (2012). Is ecotourism a viable option to generate wealth in brittle environments? A reflection on the case of the Sierra Gorda Biosphere Reserve, México. WIT Trans. Ecol. Environ..

[36] Vinson MR (2001). Long-term dynamics of an invertebrate assemblage downstream from a large dam. Ecol. Appl..

[37] Torres-Olvera MJ, Durán-Rodríguez OY, Torres-García U, Pineda-López R, Ramírez-Herrejón JP (2018). Validation of an index of biological integrity based on aquatic macroinvertebrates assemblages in two subtropical basins of central Mexico. Lat. Am. J. Aquat. Res..

[38] Carabias Lillo, J., Provencio, E., de la Maza Elvira, J. & Ruiz Corzo, M. *Programa de Manejo Reserva de la Biosfera Sierra Gorda*. (México, Instituto Nacional de Ecologıa, SEMARNAT, 1999).

[39] Macedo DR (2014). The relative influence of catchment and site variables on fish and macroinvertebrate richness in cerrado biome streams. Landsc. Ecol..

[40] Dutra SL, Callisto M (2005). Macroinvertebrates as tadpole food: Importance and body size relationships. Rev. Bras. Zool..

[41] Wang Z (2021). River-groundwater interaction affected species composition and diversity perpendicular to a regulated river in an arid riparian zone. Glob. Ecol. Conserv..

[42] López-López E, Sedeño-Díaz JE, Mendoza-Martínez E, Gómez-Ruiz A, Ramírez EM (2019). Water quality and macroinvertebrate community in dryland streams: The case of the Tehuacán-Cuicatlán Biosphere Reserve (México) facing climate change. Water (Switzerland).

[43] O’Connor NA (1991). The effects of habitat complexity on the macroinvertebrates colonising wood substrates in a lowland stream. Oecologia.

[44] Milner AM, Gloyne-Phillips IT (2005). The role of riparian vegetation and woody debris in the development of macroinvertebrate assemblages in streams. River Res. Appl..

[45] Vannote RL, Minshall GW, Cummins KW, Sedell JR, Cushing CE (1980). The river continuum concept. Can. J. Fish. Aquat. Sci..

[46] Malmqvist B, Hoffsten P-O (1999). Influence of drainage from old mine deposits on benthic macroinvertebrate communities in central Swedish streams. Water Res..

[47] Jost L (2010). Independence of alpha and beta diversities. Ecology.

[48] Cottenie K (2005). Integrating environmental and spatial processes in ecological community dynamics. Ecol. Lett..

[49] Jerves-Cobo R (2018). Biological impact assessment of sewage outfalls in the urbanized area of the Cuenca River basin (Ecuador) in two different seasons. Limnologica.

[50] US Environmental Protection Agency. *National Recommended Water Quality Criteria-Aquatic Life Criteria Table. Arsenic*. (US Environmental Protection Agency, 1995).

[51] DeNicola DM, Lellock AJ (2015). Nutrient limitation of algal periphyton in streams along an acid mine drainage gradient. J. Phycol..

[52] Younos, T. & Schreiber, M. *The Handbook of Environmental Chemistry 68. Tamim Younos, Madeline Schreiber, Katarina Kosič Ficco-Karst Water Environment-Springer International Publishing (2019).pdf*. (Springer, 2019).

[53] Robles, I. *et al.* Characterization and remediation of soils and sediments polluted with Mercury: Occurrence, transformations, environmental considerations and San Joaquin’s Sierra Gorda case. in *Environmental Risk Assessment of Soil Contamination*. 10.5772/57284. (InTech, 2014).

[54] Hernández-Silva G (2012). Presencia Del Hg total En Una Relación Suelo-Planta-Atmósfera Al Sur De La Sierra Gorda De Querétaro, México. TIP Rev. Espec. Ciencias Químico-Biol..

[55] Campos EMP, Muñoz AJH (2013). Minas y mineros: Presencia de metales en sedimentos y restos humanos al sur de la sierra gorda de Querétaro en México. Chungara.

[56] Carrillo-Martínez, M. & Suter-Cargneluti, M. Tectónica de los alrededores de Zimapán, Hidalgo y Querétaro, Libro Guía de la excursión geológica a la región de Zimapán y áreas circundantes, estados de Hidalgo y Querétaro, Hidalgo, México. in *VI Convención Geológica Nacional México, DF, Society Geológica Mexico*. 1–20. (1982).

[57] Allan JD (2007). Stream ecology: Structure and function of running waters. Stream Ecol. Struct. Funct. Run. Waters.

[58] Trang NTT, Shrestha S, Shrestha M, Datta A, Kawasaki A (2017). Evaluating the impacts of climate and land-use change on the hydrology and nutrient yield in a transboundary river basin: A case study in the 3S River Basin (Sekong, Sesan, and Srepok). Sci. Total Environ..

[59] Khatri N, Tyagi S (2015). Influences of natural and anthropogenic factors on surface and groundwater quality in rural and urban areas. Front. Life Sci..

[60] Simões NR (2015). Impact of reservoirs on zooplankton diversity and implications for the conservation of natural aquatic environments. Hydrobiologia.

[61] Dallas HF, Rivers-Moore NA (2012). Critical thermal maxima of aquatic macroinvertebrates: Towards identifying bioindicators of thermal alteration. Hydrobiologia.

[62] Struijs J, De Zwart D, Posthuma L, Leuven RS, Huijbregts MA (2011). Field sensitivity distribution of macroinvertebrates for phosphorus in inland waters. Integr. Environ. Assess. Manag..

[63] Molina CI (2010). Transfer of mercury and methylmercury along macroinvertebrate food chains in a floodplain lake of the Beni River, Bolivian Amazonia. Sci. Total Environ..

[64] Corkum LD (1989). Patterns of benthic invertebrate assemblages in rivers of northwestern North America. Freshw. Biol..

[65] Dalu T (2017). Assessing drivers of benthic macroinvertebrate community structure in African highland streams: An exploration using multivariate analysis. Sci. Total Environ..

[66] Eriksen TE (2021). A global perspective on the application of riverine macroinvertebrates as biological indicators in Africa, South-Central America, Mexico and Southern Asia. Ecol. Indic..

[67] Mercado-Garcia D (2019). Assessing the freshwater quality of a large-scale mining watershed: The need for integrated approaches. Water.

[68] Gerhardt A, Janssens De Bisthoven L, Soares AMVM (2005). Effects of acid mine drainage and acidity on the activity of *Choroterpes picteti* (Ephemeroptera: Leptophlebiidae). Arch. Environ. Contam. Toxicol..

[69] Qu X, Wu N, Tang T, Cai Q, Park Y-S (2010). Effects of heavy metals on benthic macroinvertebrate communities in high mountain streams. Ann. Limnol. Int. J. Limnol..

[70] Soucek DJ, Denson BC, Schmidt TS, Cherry DS, Zipper CE (2002). Impaired *Acroneuria* sp. (Plecoptera, Perlidae) populations associated with aluminum contamination in neutral pH surface waters. Arch. Environ. Contam. Toxicol..

[71] Ankley GT (1996). Evaluation of metal/acid-volatile sulfide relationships in the prediction of metal bioaccumulation by benthic macroinvertebrates. Environ. Toxicol. Chem..

[72] Croteau MN, Luoma SN, Stewart AR (2005). Trophic transfer of metals along freshwater food webs: Evidence of cadmium biomagnification in nature. Limnol. Oceanogr..

[73] Specht WL, Cherry DS, Lechleitner RA, Cairns J (1984). Structural, functional, and recovery responses of stream invertebrates to fly ash effluent. Can. J. Fish. Aquat. Sci..

[74] Corbi JJ, Froehlich CG, Strixino ST, Dos Santos A (2010). Bioaccumulation of metals in aquatic insects of streams located in areas with sugar cane cultivation. Quim. Nova.

[75] Poff NL, Bledsoe BP, Cuhaciyan CO (2006). Hydrologic variation with land use across the contiguous United States: Geomorphic and ecological consequences for stream ecosystems. Geomorphology.

[76] Chang FH, Lawrence JE, Rios-Touma B, Resh VH (2014). Tolerance values of benthic macroinvertebrates for stream biomonitoring: Assessment of assumptions underlying scoring systems worldwide. Environ. Monit. Assess..

[77] Brittain, J. E. Life History Strategies in Ephemeroptera and Plecoptera. in *Mayflies and Stoneflies: Life Histories and Biology*. 1–12. 10.1007/978-94-009-2397-3_1 (Springer Netherlands, 1990).

[78] Bispo PC, Oliveira LG, Bini LM, Sousa KG (2006). Ephemeroptera, Plecoptera and Trichoptera assemblages from riffles in mountain streams of central Brazil: Environmental factors influencing the distribution and abundance of immatures. Braz. J. Biol..

[79] Jacobsen, D. Tropical high-altitude streams. in *Tropical Stream Ecology*. 219–256. 10.1016/B978-012088449-0.50010-8 (Elsevier, 2008).

[80] Jacobsen D, Rostgaard S, Vasconez JJ (2003). Are macroinvertebrates in high altitude streams affected by oxygen deficiency?. Freshw. Biol..

[81] Courtney LA, Clements WH (2002). Assessing the influence of water and substratum quality on benthic macroinvertebrate communities in a metal-polluted stream: An experimental approach. Freshw. Biol..

[82] Buss DF, Salles FF (2007). Using Baetidae species as biological indicators of environmental degradation in a Brazilian river basin. Environ. Monit. Assess..

[83] Ristau K, Faupel M, Traunspurger W (2012). The effects of nutrient enrichment on a freshwater meiofaunal assemblage. Freshw. Biol..

[84] Cornelis, R. & Nordberg, M. General chemistry, sampling, analytical methods, and speciation. in *Handbook on the Toxicology of Metals*. 11–38. 10.1016/B978-012369413-3/50057-4 (Elsevier, 2007).

[85] Santore RC, Di Toro DM, Paquin PR, Allen HE, Meyer JS (2001). Biotic ligand model of the acute toxicity of metals. 2. Application to acute copper toxicity in freshwater fish and Daphnia. Environ. Toxicol. Chem..

[86] Kozlova T, Wood CM, McGeer JC (2009). The effect of water chemistry on the acute toxicity of nickel to the cladoceran Daphnia pulex and the development of a biotic ligand model. Aquat. Toxicol..

[87] Valdez R, Guzmán-Aranda JC, Abarca FJ, Tarango-Arámbula LA, Sánchez FC (2006). Wildlife conservation and management in Mexico. Wildl. Soc. Bull..

[88] INEGI. *Por Actividad Económica*. https://www.inegi.org.mx/temas/pib/. Accessed 6 Jan 2021. (2020).

[89] García, E. *Modificaciones Al Sistema de Classificación Climática de Koppen*. (Institute of Geography, UNAM, 1988).

[90] HACH. *User Manual—HACH DR 3900. in 1–148* (2013).

[91] APHA. *Standard Methods for the Examination of Water and Wastewater*. (Association, American Public Health, 2005).

[92] NMX-AA-051-SCFI-2001. Análisis de agua—Determinación de metales por absorción atómica en aguas naturales, potables, residuales y residuales tratadas. *Norma Mex.* 1–47 (2001).

[93] Helsel DR (1990). Less than obvious: Statistical treatment of data below the detection limit. Environ. Sci. Technol..

[94] Barbour MT, Stribling JB, Verdonschot PFM (2006). The multihabitat approach of USEPA’s rapid bioassessment protocols: benthic macroinvertebrates. Limnetica.

[95] USEPA. *National Rivers and Streams Assessment 2018/19: Field Operations Manual—Wadeable*. Vol. EPA-841-B-. 169. (2017).

[96] Michaud JP, Wierenga M (2005). Estimating Discharge and Stream Flows.

[97] Hering D, Moog O, Sandin L, Verdonschot PFM (2004). Overview and application of the AQEM assessment system. Hydrobiologia.

[98] Merrit R, Cummins KW (1996). An Introduction to the Aquatic Insects of North America.

[99] Thorp JH, Covich AP (2009). Ecology and Classification of North American Freshwater Invertebrates.

[100] Bueno-Soria J (2010). Guía de Identificación Ilustrada de Losgéneros de Larvas de Insectos del Orden Trichoptera de México.

[101] Springer M, Ramírez A, Hanson P (2010). Macroinvertebrados de agua dulce I. Rev. Biol. Trop..

[102] Hamada N, Thorp JH, Rogers DC (2018). Thorp and Covich’s Freshwater Invertebrates.

[103] Jost L (2010). Partitioning diversity for conservation analyses. Divers. Distrib..

[104] Lavit C, Escoufier Y, Sabatier R (1994). The ACT (STATIS method) J q G G Fl { q K q *. Comput. Stat. Data Anal..

