# Supplementary material for: Aquatic macroinvertebrate assemblages in rivers influenced by mining activities
Source: Sci Rep. 2022 Feb 25;12:3209. doi: 10.1038/s41598-022-06869-2 (PMC8881590; doi:10.1038/s41598-022-06869-2)
Supplement: Supplementary file 1 — Supplementary Tables. [file 41598_2022_6869_MOESM1_ESM.docx]

**Supplementary Material**

| **River** | **Diversity index** | **Min** | **Max** | **Mean** | **SE** |
| --- | --- | --- | --- | --- | --- |
| Extoraz | *^0^D** | 32 | 40 | 37 | 1.7 |
|  | *^1^D* | 8.3 | 11.6 | 9.2 | 0.7 |
|  | *H'* | 2.1 | 2.4 | 2.2 | 0.07 |
| Escanela-Jalpan | *^0^D** | 41 | 52 | 47 | 1.7 |
|  | *^1^D* | 3 | 13.8 | 8.9 | 1.7 |
|  | *^2^D* | 1.6 | 8.9 | 5.3 | 1.1 |
|  | *H'* | 1.1 | 2.6 | 2.1 | 0.2 |
| Concá and Ayutla | *^0^D* | 35 | 50 | 43 | 4.3 |
|  | *^1^D* | 8.4 | 14.2 | 11.2 | 1.6 |
|  | *^2^D* | 4.8 | 8.6 | 6.9 | 1.1 |
|  | *H'* | 2.1 | 2.7 | 2.4 | 0.1 |
| Santa María | *^0^D* | 44 | 51 | 47 | 3.5 |
|  | *^1^D* | 13.5 | 15.7 | 14.6 | 1 |
|  | *^2^D* | 8.7 | 11.3 | 8.7 | 1.2 |
|  | *H'* | 2.6 | 2.8 | 2.6 | 0.07 |

**Supplementary Table S1**. Summary of diversity index values. Based on all 15 sites, the minimum (min), maximum (max) and mean values, including the standard error (SE), are given for each diversity index. H’: Shannon entropy index. *0D*, *1D* and *2D*: Hill’s effective numbers. *Statistical differences by one-way ANOVA test (HSD Tukey; *p ≤* 0.

|  | Alk | Cl | Fec Col | Tot Col | Col | Cond | BOD_5_ | Hard | NH_3_ | NO_2_ | NO_3_ | TN | DO | O-PO_4_ | pH | TP | Sal |
| --- | --- | --- | --- | --- | --- | --- | --- | --- | --- | --- | --- | --- | --- | --- | --- | --- | --- |
| Alk | - | 0.3 | 0.23 | 0.14 | 0.09 | 0.37 | 0.05 | 0.41 | 0 | -0.07 | 0.13 | 0.11 | -0.41 | -0.05 | -0.23 | -0.04 | 0.35 |
| Cl |  | - | 0.36 | 0.31 | 0.09 | 0.4 | 0.21 | 0.46 | 0.17 | 0.02 | -0.04 | -0.18 | 0.08 | 0.1 | 0.25 | 0.49 | 0.36 |
| Fec Col |  |  | - | 0.92 | -0.31 | -0.34 | 0.57 | 0.13 | -0.14 | 0.08 | -0.11 | 0.03 | 0.08 | -0.09 | 0.29 | -0.08 | -0.35 |
| Tot Col |  |  | *** | - | -0.3 | -0.39 | 0.5 | 0.25 | -0.13 | 0.04 | -0.09 | -0.07 | 0.08 | -0.05 | 0.16 | -0.1 | -0.38 |
| Col |  |  |  |  | - | 0.5 | -0.18 | 0.08 | 0.91 | 0.63 | 0.49 | 0.61 | 0.15 | 0.58 | -0.29 | 0.23 | 0.47 |
| Cond |  |  |  |  |  | - | -0.52 | 0.46 | 0.37 | 0.36 | 0.36 | 0.27 | -0.43 | 0.22 | -0.52 | 0.54 | 0.99 |
| BOD_5_ |  |  |  |  |  |  | - | -0.03 | 0.05 | -0.12 | 0.03 | 0 | 0.57 | -0.1 | 0.5 | -0.24 | -0.51 |
| Hard |  |  |  |  |  |  |  | - | 0 | -0.16 | 0.15 | 0.1 | -0.14 | -0.08 | -0.24 | -0.05 | 0.49 |
| NH_3_ |  |  |  |  | *** |  |  |  | - | 0.65 | 0.58 | 0.54 | 0.28 | 0.65 | -0.24 | 0.29 | 0.36 |
| NO_2_ |  |  |  |  |  |  |  |  | * | - | 0.59 | 0.45 | 0.1 | 0.69 | -0.39 | 0.42 | 0.36 |
| NO_3_ |  |  |  |  |  |  |  |  |  |  | - | 0.14 | 0.23 | 0.53 | -0.51 | -0.07 | 0.4 |
| TN |  |  |  |  |  |  |  |  |  |  |  | - | -0.09 | 0.35 | -0.23 | 0.03 | 0.25 |
| DO |  |  |  |  |  |  |  |  |  |  |  |  | - | 0.3 | 0.39 | -0.21 | -0.45 |
| O-PO_4_ |  |  |  |  |  |  |  |  | * | * |  |  |  | - | -0.36 | 0.27 | 0.2 |
| pH |  |  |  |  |  |  |  |  |  |  |  |  |  |  | - | -0.25 | -0.54 |
| TP |  |  |  |  |  |  |  |  |  |  |  |  |  |  |  | - | 0.51 |
| Sal |  |  |  |  |  | *** |  |  |  |  |  |  |  |  |  |  | - |
| SO_4_ |  |  |  |  |  | *** |  |  |  |  |  |  |  |  |  |  | *** |
| TSS |  |  |  |  | ** |  |  |  | *** | *** |  |  |  | * |  |  |  |
| Wat Temp |  |  |  |  |  | * | * |  |  |  |  |  |  |  |  |  | * |
| Air Temp |  |  |  |  |  |  |  |  |  |  |  |  |  |  |  |  |  |
| Tur |  |  |  |  | * |  |  |  | ** | *** |  |  |  | * |  |  |  |
| Dis |  |  |  |  |  |  |  |  |  |  |  |  |  |  |  |  |  |
| Hab |  |  |  |  |  |  |  |  |  |  |  |  |  |  |  |  |  |
| Al |  |  |  |  |  |  |  |  |  |  |  |  |  | * |  |  |  |
| As |  |  |  |  |  |  |  |  |  |  |  |  |  |  |  |  |  |
| Cd |  |  |  |  |  |  |  |  |  |  |  |  |  |  |  |  |  |
| Hg |  |  |  |  |  |  |  |  |  |  |  |  |  |  | * |  |  |
| Sb |  |  |  |  |  |  |  |  |  |  |  |  |  |  |  |  |  |
| Zn |  |  |  |  |  |  |  |  |  |  |  |  |  |  |  |  |  |
| Co |  |  |  |  |  |  |  |  |  |  |  |  |  |  |  |  |  |
| Cr |  |  |  |  |  |  |  |  |  |  |  |  |  |  |  |  |  |
| Cu |  |  |  |  |  |  |  |  |  |  |  |  |  |  |  |  |  |
| Fe |  |  |  |  |  |  |  |  |  |  |  |  |  | * |  |  |  |
|  |  |  |  |  |  |  |  |  |  |  |  |  |  |  |  |  |  |

**Supplementary Table S2.** Collinearity between predictors: correlations (Spearman’s rho) are shown above the diagonal, and p-values are shown below the diagonal. The level of significance has been adjusted according to the high number of pairwise comparisons. ****p* < 0.0001, ***p* < 0.001, **p* < 0.01. Bold: Predictors that showed high collinearity (Spearman’s rho > 0.80) and were consequently excluded from further analyses. Abbreviations are explained in Supplementary Table S3.

|  | SO_4_ | TSS* | Water Temp | Air Temp | Tur* | Dis | Hab | Al | As | Cd | Hg | Sb | Zn | Co | Cr | Cu | Fe |
| --- | --- | --- | --- | --- | --- | --- | --- | --- | --- | --- | --- | --- | --- | --- | --- | --- | --- |
| Alk | 0.05 | -0.12 | 0.17 | 0.16 | -0.11 | -0.53 | -0.37 | -0.15 | 0.41 | 0.56 | 0.33 | 0.14 | 0.58 | 0.06 | 0.06 | 0.02 | -0.04 |
| Cl | 0.44 | 0.14 | 0 | 0 | 0.13 | -0.45 | 0 | -0.14 | -0.2 | -0.06 | -0.17 | -0.25 | 0.28 | -0.43 | -0.43 | -0.13 | -0.05 |
| Fec Col | -0.25 | -0.08 | -0.59 | -0.4 | -0.02 | -0.46 | -0.25 | -0.49 | 0.45 | 0.1 | -0.2 | 0.15 | 0.36 | 0.19 | 0.19 | 0.18 | -0.42 |
| Tot Col | -0.26 | -0.13 | -0.59 | -0.35 | -0.14 | -0.56 | -0.38 | -0.39 | 0.35 | 0.18 | -0.09 | 0.02 | 0.4 | 0.37 | 0.37 | 0.37 | -0.34 |
| Col | 0.54 | 0.81 | 0.43 | 0.45 | 0.73 | 0.25 | -0.07 | 0.59 | 0.07 | 0.25 | 0.53 | -0.11 | -0.1 | -0.16 | -0.16 | -0.15 | 0.6 |
| Cond | 0.86 | 0.54 | 0.74 | 0.22 | 0.39 | 0.09 | 0.11 | 0.36 | -0.08 | 0.2 | 0.5 | -0.38 | -0.01 | -0.25 | -0.25 | -0.46 | 0.48 |
| BOD_5_ | -0.45 | -0.18 | -0.7 | -0.09 | 0 | -0.17 | 0.04 | -0.62 | 0.01 | 0.05 | -0.49 | 0.11 | 0.12 | -0.37 | -0.37 | 0.05 | -0.59 |
| Hard | 0.38 | 0.04 | 0.21 | -0.2 | -0.25 | -0.54 | -0.06 | -0.05 | -0.05 | 0.36 | 0.44 | -0.33 | 0.43 | 0.06 | 0.06 | 0.16 | 0.12 |
| NH_3_ | 0.43 | 0.84 | 0.26 | 0.43 | 0.8 | 0.23 | -0.12 | 0.49 | -0.08 | 0.24 | 0.38 | -0.3 | -0.08 | -0.25 | -0.25 | -0.13 | 0.53 |
| NO_2_ | 0.48 | 0.85 | 0.41 | 0.4 | 0.84 | 0.48 | -0.16 | 0.43 | 0.3 | 0.02 | 0.4 | -0.27 | -0.13 | -0.09 | -0.09 | -0.45 | 0.44 |
| NO_3_ | 0.32 | 0.59 | 0.36 | 0.21 | 0.51 | 0.34 | -0.03 | 0.3 | -0.08 | 0.35 | 0.37 | -0.43 | 0.29 | -0.19 | -0.19 | -0.05 | 0.43 |
| TN | 0.31 | 0.62 | 0.18 | 0.09 | 0.51 | 0.12 | -0.03 | 0.32 | 0.32 | 0.26 | 0.53 | 0.04 | -0.17 | 0.06 | 0.06 | -0.28 | 0.38 |
| DO | -0.23 | 0.1 | -0.29 | 0.15 | 0.19 | 0.22 | 0.33 | -0.17 | -0.25 | -0.46 | -0.37 | -0.09 | -0.08 | -0.43 | -0.43 | 0.04 | -0.2 |
| O-PO_4_ | 0.31 | 0.69 | 0.48 | 0.53 | 0.65 | 0.19 | -0.21 | 0.66 | -0.17 | -0.1 | 0.39 | -0.49 | 0.22 | -0.12 | -0.12 | -0.2 | 0.71 |
| pH | -0.28 | -0.39 | -0.62 | -0.28 | -0.28 | -0.2 | 0.11 | -0.33 | -0.06 | -0.28 | -0.76 | 0.45 | -0.14 | -0.37 | -0.37 | 0.09 | -0.47 |
| TP | 0.47 | 0.42 | 0.42 | 0.47 | 0.51 | 0.21 | -0.03 | 0.13 | -0.12 | -0.28 | 0.09 | -0.43 | -0.35 | -0.31 | -0.31 | -0.62 | 0.15 |
| Sal | 0.85 | 0.54 | 0.75 | 0.2 | 0.36 | 0.1 | 0.07 | 0.37 | -0.11 | 0.26 | 0.52 | -0.43 | 0.01 | -0.25 | -0.25 | -0.44 | 0.5 |
| SO_4_ | - | 0.65 | 0.55 | 0.06 | 0.43 | 0.11 | 0.22 | 0.48 | -0.18 | 0.04 | 0.3 | -0.34 | -0.12 | -0.28 | -0.28 | -0.43 | 0.53 |
| TSS | * | - | 0.46 | 0.24 | 0.91 | 0.43 | 0.01 | 0.55 | 0.03 | 0.1 | 0.49 | -0.37 | -0.13 | -0.19 | -0.19 | -0.35 | 0.64 |
| Water Temp |  |  | - | 0.52 | 0.34 | 0.33 | -0.09 | 0.62 | -0.09 | 0 | 0.65 | -0.46 | 0.02 | -0.12 | -0.12 | -0.44 | 0.68 |
| Air Temp |  |  |  | - | 0.37 | 0.24 | -0.31 | 0.32 | -0.03 | -0.1 | 0.25 | -0.32 | -0.17 | -0.31 | -0.31 | -0.45 | 0.23 |
| Tur |  | *** |  |  | - | 0.56 | 0.05 | 0.35 | 0.1 | -0.03 | 0.29 | -0.22 | -0.19 | -0.31 | -0.31 | -0.45 | 0.43 |
| Dis |  |  |  |  |  | - | 0.41 | 0.15 | -0.04 | -0.33 | 0 | -0.05 | -0.56 | -0.31 | -0.31 | -0.48 | 0.12 |
| Hab |  |  |  |  |  |  | - | -0.31 | -0.33 | -0.55 | -0.39 | 0.1 | -0.41 | -0.37 | -0.37 | -0.27 | -0.26 |
| Al |  |  |  |  |  |  |  | - | -0.22 | 0.14 | 0.51 | -0.3 | 0.06 | 0.12 | 0.12 | -0.01 | 0.95 |
| As |  |  |  |  |  |  |  |  | - | 0.27 | 0.31 | 0.56 | 0.07 | 0.43 | 0.43 | 0.01 | -0.25 |
| Cd |  |  |  |  |  |  |  |  |  | - | 0.47 | 0.1 | 0.46 | 0.29 | 0.29 | 0.38 | 0.23 |
| Hg |  |  | * |  |  |  |  |  |  |  | - | -0.25 | 0.25 | 0.44 | 0.44 | 0.06 | 0.64 |
| Sb |  |  |  |  |  |  |  |  |  |  |  | - | -0.01 | 0.26 | 0.26 | 0.28 | -0.4 |
| Zn |  |  |  |  |  |  |  |  |  |  |  |  | - | 0.25 | 0.25 | 0.52 | 0.22 |
| Co |  |  |  |  |  |  |  |  |  |  |  |  |  | - | 1 | 0.62 | 0.12 |
| Cr |  |  |  |  |  |  |  |  |  |  |  |  |  | *** | - | 0.62 | 0.12 |
| Cu |  |  |  |  |  |  |  |  |  |  |  |  |  |  |  | - | 0.02 |
| Fe |  | * | * |  |  |  |  | *** |  |  |  |  |  |  |  |  | - |

|  | Sites | Al | As | Cd | Hg | Sb | Zn | Co | Cr | Cu | Fe | Mn |
| --- | --- | --- | --- | --- | --- | --- | --- | --- | --- | --- | --- | --- |
| Extoraz  river | PB | 2.179 | 0.027 | 0.013 | 0.020 | 0.001 | 0.227 | 0.001 | 0.001 | 0.001 | 0.825 | 0.035 |
|  | EP | 0.004 | 0.031 | 0.277 | 0.016 | 0.001 | 0.034 | 0.001 | 0.001 | 0.001 | 0.063 | 0.001 |
|  | RQ | 0.090 | 0.022 | 0.001 | 0.012 | 0.001 | 0.086 | 0.001 | 0.001 | 0.001 | 0.138 | 0.001 |
|  | BC | 0.044 | 0.024 | 0.001 | 0.003 | 0.031 | 0.005 | 0.001 | 0.001 | 0.001 | 0.044 | 0.001 |
| Escanela  river | ES | 0.037 | 0.018 | 0.001 | 0.003 | 0.009 | 0.538 | 0.001 | 0.001 | 0.003 | 0.070 | 0.001 |
|  | EN | 0.001 | 0.021 | 0.001 | 0.003 | 0.008 | 0.022 | 0.001 | 0.001 | 0.001 | 0.030 | 0.001 |
|  | AH | 0.050 | 0.046 | 0.001 | 0.003 | 0.002 | 0.020 | 0.001 | 0.001 | 0.001 | 0.037 | 0.001 |
| Jalpan  river | PI | 0.114 | 0.002 | 0.046 | 0.004 | 0.002 | 0.068 | 0.001 | 0.001 | 0.008 | 0.136 | 0.001 |
|  | JL | 0.175 | 0.085 | 0.041 | 0.058 | 0.013 | 0.080 | 0.003 | 0.004 | 0.012 | 0.190 | 0.001 |
|  | PA | 0.101 | 0.002 | 0.001 | 0.003 | 0.001 | 0.025 | 0.001 | 0.001 | 0.001 | 0.122 | 0.001 |
| Ayutla  river | AY | 0.031 | 0.044 | 0.001 | 0.005 | 0.014 | 0.021 | 0.001 | 0.001 | 0.001 | 0.034 | 0.001 |
| Concá  river | VC | 0.439 | 0.017 | 0.001 | 0.055 | 0.001 | 0.012 | 0.001 | 0.001 | 0.001 | 0.258 | 0.001 |
|  | CN | 0.528 | 0.003 | 0.001 | 0.006 | 0.001 | 0.008 | 0.001 | 0.001 | 0.001 | 0.303 | 0.001 |
| Santa  María river | SM | 0.308 | 0.042 | 0.044 | 0.007 | 0.023 | 0.059 | 0.001 | 0.001 | 0.001 | 0.212 | 0.001 |
|  | AT | 0.272 | 0.002 | 0.001 | 0.004 | 0.001 | 0.025 | 0.001 | 0.001 | 0.001 | 0.197 | 0.001 |

**Supplementary Table S3**. Metal concentrations (mg L^-1^) recorded in each sampling site and river in the SGBR.

| Data | Selected variables for abundance | *r* |
| --- | --- | --- |
| Entire data | water and air temperature, total phosphorous, and mercury | 0.591329 |
| The best model has four parameter (max. 26 allowed); correlations Spearman | | |

**Supplementary Table S4**. BIOENV results from correlations between environmental variables and macroinvertebrate assemblage abundance in the SGBR.

| **Factor** | **Unit** | **Factor** | **Unit** |
| --- | --- | --- | --- |
| Ammonia (NH3) | mg L-1 | Total coliforms (Tot Col) | MPN/100 ml |
| Nitrites (NO2) | mg L-1 | Discharge (Dis) | m3 s-1 |
| Nitrates (NO3) | mg L-1 | Habitat quality (Habitat) | 0-20 |
| Total nitrogen (TN) | mg L-1 | Fauna suitability (Fau) | 0-20 |
| Orthophosphates (O-PO4) | mg L-1 | Substrate suitability (Subs) | 0-20 |
| Total phosphorous (TP) | mg L-1 | Velocity water (Vel) | 0-20 |
| Biochemical oxygen demand (BOD5) | mg L-1 | Sediment herogeneity (Sedim) | 0-20 |
| Dissolved oxygen (DO) | mg L-1 | Channel width (Chann) | 0-20 |
| pH | - | Channel alteration (Chann alt) | 0-20 |
| Turbidity (Tur) | NTU | Channel sinuisity (Chann sinu) | 0-20 |
| Water Temperature (Water Temp) | °C | Bank availability (Bank) | 0-20 |
| Air Temperature (Air Temp) | °C | Vegetation protection cover (Veg) | 0-20 |
| Acid neutralizer Alkalinity (Alk) | CaCO3 | Riparian vegetation cover (Rip) | 0-20 |
| Total hardness (Hard) | CaCO3 | Aluminum (Al) | mg L-1 |
| Conductivity (Cond) | mS cm-1 | Antimony (Sb) | mg L-1 |
| Chlorides (Cl) | mg L-1 | Arsenic (As) | mg L-1 |
| Salinity (Sal) | PSU | Cadmium (Cd) | mg L-1 |
| Total suspended solids (TSS) | mg L-1 | Chromium (Cr) | mg L-1 |
| Sulfates (SO4) | mg L-1 | Copper (Cu) | mg L-1 |
| Color (Col) | Pt-Co | Mercury (Hg) | mg L-1 |
| Fecal coliforms (Fec Col) | MPN/100 ml | Zinc (Zn) | mg L-1 |

**Supplementary Table S5**: Environmental factors analyzed and corresponding units.
